# Endorsement of the CONSORT Statement by High-Impact Medical Journals in China: A Survey of Instructions for Authors and Published Papers

**DOI:** 10.1371/journal.pone.0030683

**Published:** 2012-02-13

**Authors:** Xiao-qian Li, Kun-ming Tao, Qing-hui Zhou, David Moher, Hong-yun Chen, Fu-zhe Wang, Chang-quan Ling

**Affiliations:** 1 Department of Traditional Chinese Medicine, Second Military Medical University, Changhai Hospital, Shanghai, China; 2 Department of Anesthesiology, Second Military Medical University, Eastern Hepatobiliary Surgical Hospital, Shanghai, China; 3 Clinical Epidemiology Program, Ottawa Hospital Research Institute, Ottawa, Canada; 4 Shanghai Municipal Education Commission, E-Institute of Traditional Chinese Internal Medicine, Shanghai University of Traditional Chinese Medicine, Shanghai, China; University of Illinois-Chicago, United States of America

## Abstract

**Background:**

The CONSORT Statement is a reporting guideline for authors when reporting randomized controlled trials (RCTs). It offers a standard way for authors to prepare RCT reports. It has been endorsed by many high-impact medical journals and by international editorial groups. This study was conducted to assess the endorsement of the CONSORT Statement by high-impact medical journals in China by reviewing their instructions for authors.

**Methodology/Principal Findings:**

A total of 200 medical journals were selected according to the Chinese Science and Technology Journal Citation Reports, 195 of which publish clinical research papers. Their instructions for authors were reviewed and all texts mentioning the CONSORT Statement or CONSORT extension papers were extracted. Any mention of the Uniform Requirements for Manuscripts Submitted to Biomedical Journals (URM) developed by the International Committee of Medical Journal Editors (ICMJE) or ‘clinical trial registration’ was also extracted. For journals endorsing the CONSORT Statement, their most recently published RCT reports were retrieved and evaluated to assess whether the journals have followed what the CONSORT Statement required. Out of the 195 medical journals publishing clinical research papers, only six (6/195, 3.08%) mentioned ‘CONSORT’ in their instructions for authors; out of the 200 medical journals surveyed, only 14 (14/200, 7.00%) mentioned ‘ICMJE’ or ‘URM’ in their instructions for authors, and another five journals stated in their instructions for authors that clinical trials should have trial registration numbers and that priority would be given to clinical trials which had been registered. Among the 62 RCT reports published in the six journals endorsing the CONSORT Statement, 20 (20/62, 32.26%) contained flow diagrams and only three (3/62, 4.84%) provided trial registration information.

**Conclusions/Significance:**

Medical journals in China endorsing either the CONSORT Statement or the ICMJE's URM constituted a small percentage of the total; all of these journals used ambiguous language regarding what was expected of authors.

## Introduction

In 1996, in response to concerns about quality of reporting of randomised controlled trials (RCTs), an international group developed the Consolidated Standards of Reporting Trials (CONSORT) Statement [1]. As a worldwide accepted guideline for reporting RCTs, the CONSORT Statement has been in use for 15 years and was updated twice in 2001 and 2010, respectively [2], [3]. The CONSORT Statement, which is an evidence-based, minimum set of recommendations for reporting RCTs, offers a standard way for authors to prepare reports of trial findings, facilitating their complete and transparent reporting, and aiding their critical appraisal and interpretation [4], [5]. CONSORT has been endorsed by several hundred medical journals, including many high-impact-factor ones, and by international editorial groups, such as the International Committee of Medical Journal Editors (ICMJE). Since the publication of CONSORT, several studies have been conducted to assess the impact of using the CONSORT Statement on improving the reporting of RCTs in medical journal articles [6]–[8]. The majority of their results indicated that CONSORT has helped improve the quality of reporting of RCTs and thus confirm its usefulness.

The ICMJE developed the Uniform Requirements for Manuscripts Submitted to Biomedical Journals (URM) primarily to help authors and editors in their mutual task of creating and distributing accurate, clear, easily accessible reports of biomedical studies. The ICMJE states in URM the ethical principles in the conduct and reporting of research and provides recommendations relating to specific elements of editing and writing [9]. Also, in order to urge researchers to conduct research ethically and to report it honestly, in 2004, the members of the ICMJE published a joint editorial aimed at promoting registration of all clinical trials and stated that ICMJE member journals will consider a trial for publication only if it has been registered before the enrollment of the first patient [10].

According to the Global Research Report released by Thomson Reuters in 2009 [11], China produced approximately 400,000 papers in all fields of science in a recent five-year period, accounting for approximately 8·5% of the world's papers published in journals indexed by Thomson Reuters. Thomson Reuters recorded 1,745 papers from China in 1981 but 127,075 in 2009, representing an astonishing change in world share from 0·4% to 10·9%. China is now second to the US, the largest single-nation producer, and will likely surpass the EU27 nations in research paper output in the next decade [12]. Also, there are over 1,000 medical journals published in China now and this number is still rising. Although most of them have not ranked as the world leading journals in their specialties, some of them have already been adhering to international conventions.

Since the quality of clinical medical research papers from Chinese authors has always been a topic of discussion and the guiding impact of medical journals should not be ignored, it is necessary to get an overview of the Chinese medical journals' requirements for their authors. Hence in this study, the endorsement of the CONSORT Statement by high-impact medical journals in China in their instructions for authors was investigated, and any mention of the ICMJE's URM was also studied.

## Methods

From April 2011 to June 2011, using the 2009 impact factors of the Chinese Science and Technology Journal Citation Reports (CSTJCR) released by the Institute of Science and Technical Information of China [13], the top 20 journals of each of the four categories including internal medicine, surgery, clinical medicine, and Chinese medicine and top ten journals of each of the other 12 categories (oncology; healthcare medicine; gynaecology, obstetrics and pediatrics; nursing; medical imaging; stomatology; neurology and psychiatry; ophthalmology and otorhinolaryngology; pharmacy; medical university journals; general medicine; preventive medicine and hygienics) were selected according to different total number of each category. A total of 200 high-impact medical journals were identified and their ‘instructions for authors’ were downloaded from their websites or copied from the most recently published issues. Two authors (Li XQ and Tao KM) read these instructions and extracted text mentioning ‘CONSORT’, ‘ICMJE’, ‘URM’, or ‘trial registration’, independently. A third author (Zhou QH) was consulted if consensus could not be reached between the two authors. The texts mentioning the above words were analyzed to evaluate the strength of their requirements for authors. By reading these instructions and browsing the published articles of the last few years in databases or journal websites, those journals that do not publish clinical trials were excluded when judging the endorsement of the CONSORT Statement.

Full-text papers of RCTs published in the most recent issues (June 2010 to June 2011 for monthly or bimonthly published journals and January 2011 to June 2011 for those published semimonthly or weekly) of the journals which mentioned ‘CONSORT’ in their instructions for authors were downloaded. Two authors (Li XQ and Tao KM) reviewed these RCT reports to see whether they contained flow diagrams as the CONSORT Statement required, and whether they provided trial registration information.

## Results

### Endorsement of the CONSORT Statement by high-impact medical journals in China

By reading instructions for authors and by browsing the published articles in databases or journals' websites, those journals that do not publish clinical trials were excluded (*n* = 5). Out of the remaining 195 journals, only six mentioned ‘CONSORT’ in their instructions for authors (6/195, 3.08%). Among the six, two gave references which published the CONSORT Statement (either English version or Chinese version) as well as web links to the CONSORT website; these two journals also listed several other reporting standards such as TREND, PRISMA, etc. Although some of them are not newly updated, the web links may ensure accurate reference for their readers or authors. One journal gave a web link to the CONSORT website and a 22-item checklist of the 2001 version. Two journals stated that authors of RCTs should provide the CONSORT checklist and a flow chart of the trial. One recommended authors of RCTs to refer to the CONSORT Statement. Those journals that did not mention CONSORT provided no other reporting guidance for authors.

### Endorsement of the URM by high-impact medical journals in China

Out of the 200 journals surveyed, only 14 journals mentioned ‘ICMJE’ or ‘URM’ in their instructions for authors (14/200, 7.00%). They either gave the ICMJE website link, or gave a reference to a published URM text, or mentioned an exact version they endorsed, or quoted some texts from the URM, or specially mentioned trial registration procedures. Apart from these 14 journals, five journals stated in their instructions for authors that clinical trials should have trial registration numbers and that priority would be given to clinical trials which had been registered.

### RCT reports from the journals endorsing the CONSORT Statement

A total of 62 RCT reports were collected from the six journals endorsing the CONSORT Statement. They were all published in the most recent issues. Among these RCT reports, 20 (20/62, 32.26%) contained flow diagrams and only three (3/62, 4.84%) provided trial registration information (Table 1).

**Table 1:** RCT reports from 6 Chinese medical journals endorsing the CONSORT Statement.

Journal title	Publication frequency	Journal issues reviewed	Numbers of RCT reports		
			Collected	Containing a flow diagram	Providing trial registration information
*Chinese Journal of Cancer Biotherapy*	Bimonthly	June 2010 to June 2011	2	0	0
*Chinese Journal of Evidence-Based Medicine*	Monthly	June 2010 to June 2011	5	1	0
*Chinese Journal of Evidence-based Pediatrics*	Bimonthly	June 2010 to June 2011	3	1	0
*Chinese Medical Journal* (English version)	Semimonthly	January 2011 to June 2011	23	5	3
*Journal of Chinese Integrative Medicine*	Monthly	June 2010 to June 2011	10	10	0
*Journal of Clinical Rehabilitative Tissue Engineering Research*	Weekly	January 2011 to June 2011	19	3	0
Total	—	—	62	20	3

RCT: randomised controlled trial.

## Discussion

The CSTJCR is the most authoritative citation reports in China, which is released by the Institute of Science and Technical Information of China and updated once a year [13]. Journals indexed in this citation report are called ‘core journals’ and have achieved a good reputation in China. Chinese authors consistently select these journals for publication of their research papers as well as degree papers. In this study, 200 core journals were selected from 16 medical specialty categories in the 2009 citation reports for investigating the endorsement of the CONSORT Statement and the URM by these high-impact medical journals in China. There are only six journals that mentioned ‘CONSORT’ in their instructions for authors and the number of journals that mentioned the ICMJE or the URM is slightly more at 14; both constitute a small percentage of the total. The two proportions are much lower than those reported in Altman's study which selected journals indexed in the Science Citation Index (SCI) [6]. Five additional journals stated in their instructions for authors that clinical trials should have trial registration numbers and priority will be given to clinical trials which had been registered. However, all journals endorsing either the CONSORT Statement or the URM used ambiguous language regarding what was expected of authors.

RCT reports published in the most recent issues of journals that endorsed the CONSORT Statement were reviewed. As the participant flow diagram is strongly recommended in the CONSORT 2010 Statement [3], we chose the flow diagram as the standard to assess whether the journal had followed the CONSORT Statement requirements. By reviewing the 62 published RCTs in the six journals endorsing the CONSORT Statement, only one third (20/62, 32.26%) provided flow diagrams. It could be speculated that inclusion of flow diagrams in RCT papers was dependent on whether the authors have provided one. Only the *Journal of Chinese Integrative Medicine* is an exception as each of the ten RCTs published in its past one year issues included a flow diagram. For some randomized trials the flow of participants through each phase of the trial can be relatively straightforward to describe, particularly where there were no losses to follow-up or exclusions. However, in more complex trials it may be difficult for readers to discern whether and why some participants did not receive the treatment as allocated, were lost to follow-up, or were excluded from the analysis [14]. A completed flow diagram is especially valuable for such trials. A complete CONSORT flow diagram reduces the time taken for readers to find the essential information to assess the reliability of a trial. It is also likely to improve the availability of some information which otherwise might not be reported. Poor reporting of participant eligibility makes it difficult to know whether the enrolment process was highly selective and whether those who enrolled were representative of the general population [15], [16].

‘Trial registration number and name of trial registry’ is a new item added in the CONSORT 2010 checklist as empirical evidence supports the need for trial registration, and requirements by ICMJE in 2004 have fostered compliance [3], [10]. So whether trial registration information is incorporated in these published RCTs was also judged as a standard. Only three RCT papers provided trial registration information, which were all published in the *Chinese Medical Journal* (English version), taking a small percentage of all its published RCTs between January 2011 to June 2011 (3/23, 13.04%).

Among the 200 Chinese medical journals, four journals are indexed in the SCI or SCI-E. However, among these four journals, only one journal endorsed the CONSORT Statement and only two journals gave a web link to the ICMJE website. The SCI journals are deemed as representative of high-quality and high-impact journals; nevertheless, the endorsement of the CONSORT Statement or the URM by these Chinese SCI journals is not encouraging. Also, by reviewing the instructions for authors of the series journals of the Chinese Medical Association, which are regarded as the high-quality standard for Chinese medical journals, none of them except the *Chinese Medical Journal* (English version) mentioned the CONSORT Statement or gave a web link to the ICMJE website.

The CONSORT Statement and the URM as well as their revised or updated versions have already been translated into Chinese and published in some Chinese medical journals [17]–[25]. Therefore it is assumed that journal editors as well as authors in China could have easy access to the CONSORT Statement and the URM which are easily intelligible to them. It is noteworthy to mention that those journals that lack CONSORT did not use any alternative guideline to the CONSORT Statement, namely, they simply lack guidelines.

A number of clinical research papers are published in China each year; however, according to several studies evaluating the quality of reporting of RCTs conducted in China, none of them achieved positive results [26]–[28]. When looking back to the vehicle of these papers, as what is indicated in this study, we may see why the quality of clinical research papers in China lag behind so far from the international standard. The reasons that so few medical journals in China endorsed the CONSORT Statement or the URM in their instructions for authors are assumed as follows. First, most medical schools, hospitals or medical research institutes in China required their fellows to publish their papers in the core journals to facilitate their promotion or graduation, but with a lack of attention paid to who conducted what aspects of the study. Within such a bureaucratic system, the focus is on quick success and instant benefits. On the other hand, core medical journals are in large quantities and the good and the bad are intermingled. So if one journal enhances the threshold for its acceptance of manuscripts, it would lose most authors and obtain little benefit. After all, not all journals in China have the need to be adhering to international conventions. Second, Chinese authors who conducted high-quality clinical research, would prefer to publish their papers in high-impact SCI journals instead of domestic core journals. As a result, the low-quality journals and the low-quality manuscripts have created a chicken-and-egg problem in China. So even for some international journals in China such as those endorseing the CONSORT Statement, the URM, or trial registration in their instructions for authors, they would not tend to stress these requirements to their authors. This explained why we found that most of the six journals endorsing the CONSORT Statement failed to adhere to the standards by reviewing their published RCTs.

This work has some limitations. First, this study used the electronic resources which may change during the study period. However, at the end of the study, no journals updated their instructions for authors. Second, no telephone calls were made and no emails were sent to the editorial offices of the journals investigated as per the study by Hopewell *et al* [8]. Therefore, we do not know the opinions of the journal editors about the issue concerned in this study. However, having reviewed the published papers in these journals, it could be estimated that their responses would not be positive. Third, some Chinese medical journals that are indexed in the SCI but not in the CSTJCR due to few citations by Chinese journals were not included in this study. The number of such journals is very small so we may assume that the results would not be affected.
